# Sequence polymorphism of the *waxy* gene in waxy maize accessions and characterization of a new *waxy* allele

**DOI:** 10.1038/s41598-020-72764-3

**Published:** 2020-09-28

**Authors:** Meijie Luo, Yaxing Shi, Yang Yang, Yanxin Zhao, Yunxia Zhang, Yamin Shi, Mengsi Kong, Chunhui Li, Zhen Feng, Yanli Fan, Li Xu, Shengli Xi, Baishan Lu, Jiuran Zhao

**Affiliations:** Beijing Key Laboratory of Maize DNA Fingerprinting and Molecular Breeding, Maize Research Center, Beijing Academy of Agriculture and Forestry Sciences (BAAFS), Beijing, 100097 China

**Keywords:** Plant breeding, PCR-based techniques

## Abstract

Waxy maize has many excellent characteristics in terms of its nutritional and economic value. In recent decades, the waxy maize germplasm has increased dramatically as a result of different selection methods. We collected 200 waxy maize inbred accessions from different origins to study their genetic diversity and phylogenetic relationships, and to identify new *waxy* mutations. A simple sequence repeat (SSR) analysis revealed wide genetic diversity among the 200 waxy maize accessions. The maize accessions were clustered into three groups. We sequenced the *waxy* gene from the first to the 14th exon. Nucleotide variation analysis of 167 waxy maize and 14 flint maize lines revealed some nucleotide differences in the *waxy* gene among different waxy maize groups, and much narrower nucleotide diversity in waxy maize than in flint maize. In a phylogenetic analysis, waxy maize carrying the same mutation allele clustered together, and waxy maize carrying different mutation alleles distributed in different groups; waxy maize was intermixed with flint maize in each branch, and *wx-D7* waxy maize separated significantly from waxy maize lines carrying *wx-D10, wx-124* and *wx-hAT* mutant alleles. The *wx-hAT* was a new *waxy* mutation identified in this study. It consisted of a 2286-bp transposon inserted into the middle of exon three of the *waxy* gene. A PCR marker specific for the *wx-hAT* allele was developed. These results will be useful for the utilization and preservation of the waxy maize germplasm, and the PCR marker has potential uses in waxy maize breeding programs.

## Introduction

Waxy maize, also known as sticky maize, has high economic, nutritional, and processing value^1,2^. The starch in the endosperm of waxy maize is nearly 100% amylopectin, which confers the sticky quality to maize grains. It is mainly consumed as food in Asia, and is also an important ingredient in the textile and paper industries. Waxy maize was first discovered in China in 1908, and was later reported in other locations of Asia^2^. The results of several studies suggest that the southwest region of China, particularly Yunnan Province and its surrounding areas, is the central origin of Chines waxy maize^3,4^. In recent decades, many adapted maize lines have been developed for hybrid seed production by different selection methods, but the relationships among these waxy maize lines are unclear.

The glutinous genotypes of waxy maize are null mutations of the *waxy* gene, which encodes the granule bound starch synthase (GBSSI) that is necessary for amylose synthesis^5–7^. The wild-type *waxy* gene in maize is 3.93 kb long, located on chromosome 9, and composed of 14 exons. Insertions and deletions in the DNA sequence are the main types of *waxy* mutations in maize^8^. Globally, more than 50 mutations in the *waxy* gene have been characterized at the molecular level^8^. In Chinese waxy maize, the two deletion mutations *wx-D7* and *wx-D10* (a 30-bp deletion in the seventh exon and a 15-bp deletion in the tenth exon, respectively) are the main *waxy* alleles^9,10^. Transposable element insertions are another type of *waxy* gene mutation^8^. Transposons can be divided into DNA transposons and RNA transposons according to their transposition mechanism. Members of the DNA-transposon family can jump from one gene to another in a cut-and-paste fashion to produce unstable mutants^11,12^. Among the identified mutations in the maize *waxy* gene, *wx-m9*, *wx-m5*, *wx-B3*, *wx-m1*, *wx-B4*, *wx-m6*, and *wx-m7* are insertion mutations of Ac/Ds transposable elements, *wx-m8* is an insertion mutation of the dSpm element, and *wx-844* is a mutation caused by the En/Spm element^8,11^. The Ac/Ds, dSpm, and En/Spm elements are all members of the DNA-transposon family^12^ and their *waxy* alleles are important resources for transposon research. RNA transposons move within the genome using a copy-and-paste mechanism via reverse transcription of an RNA intermediate^3^. Among the known mutant alleles of the *waxy* gene in maize, *wx-stonor*, *wx-B5*, *wx-G*, *wx-M*, *wx-I*, *wx-K*, *wx-Cin4* and *wx-Rina* are RNA transposon insertion mutations^3,8^. RNA transposons result in stable mutants and can be used as markers to identify *waxy* mutation loci. However, there are still many waxy maize lines with unknown *waxy* alleles.

There is wide genetic diversity at the *waxy* locus among waxy maize accessions. Previous studies on *waxy* diversity have shown that different *waxy* mutants have independent origins^10^. Compared with non-glutinous maize, Chinese waxy maize shows much narrower nucleotide diversity at the *waxy* locus, suggesting that there has been strong selection in the *waxy* genomic region during waxy maize breeding^13,14^. An association analysis between allelic variations of *waxy* and starch physicochemical properties showed that *waxy* allelic variation affects the gel consistency, gelatinization temperature, and pasting viscosity properties of rice starch, implying that certain *waxy* alleles have been favored for grain quality improvement^15,16^. Therefore, comparison of *waxy* sequence variation among maize germplasm accessions not only provides insights into selection at the maize *waxy* locus during domestication, but also can highlight mutations in the *waxy* gene that will be useful for the germplasm utilization and quality breeding of waxy maize^16,17^.

In this study, we collected waxy maize breeding accessions from Jilin province, Shanxi province, and Beijing in China, as well as from Korea. First, we used simple sequence repeat (SSR) markers to study the genetic diversity of the waxy maize inbred lines. Then we sequenced the *waxy* genes from the first to the fourteenth exon to analyze sequence variations and the phylogenetic relationships among waxy maize lines. Additionally, a newly identified allele of the *waxy* gene in waxy maize was characterized.

## Results

### Genetic analyses of waxy maize

**Figure 1:**
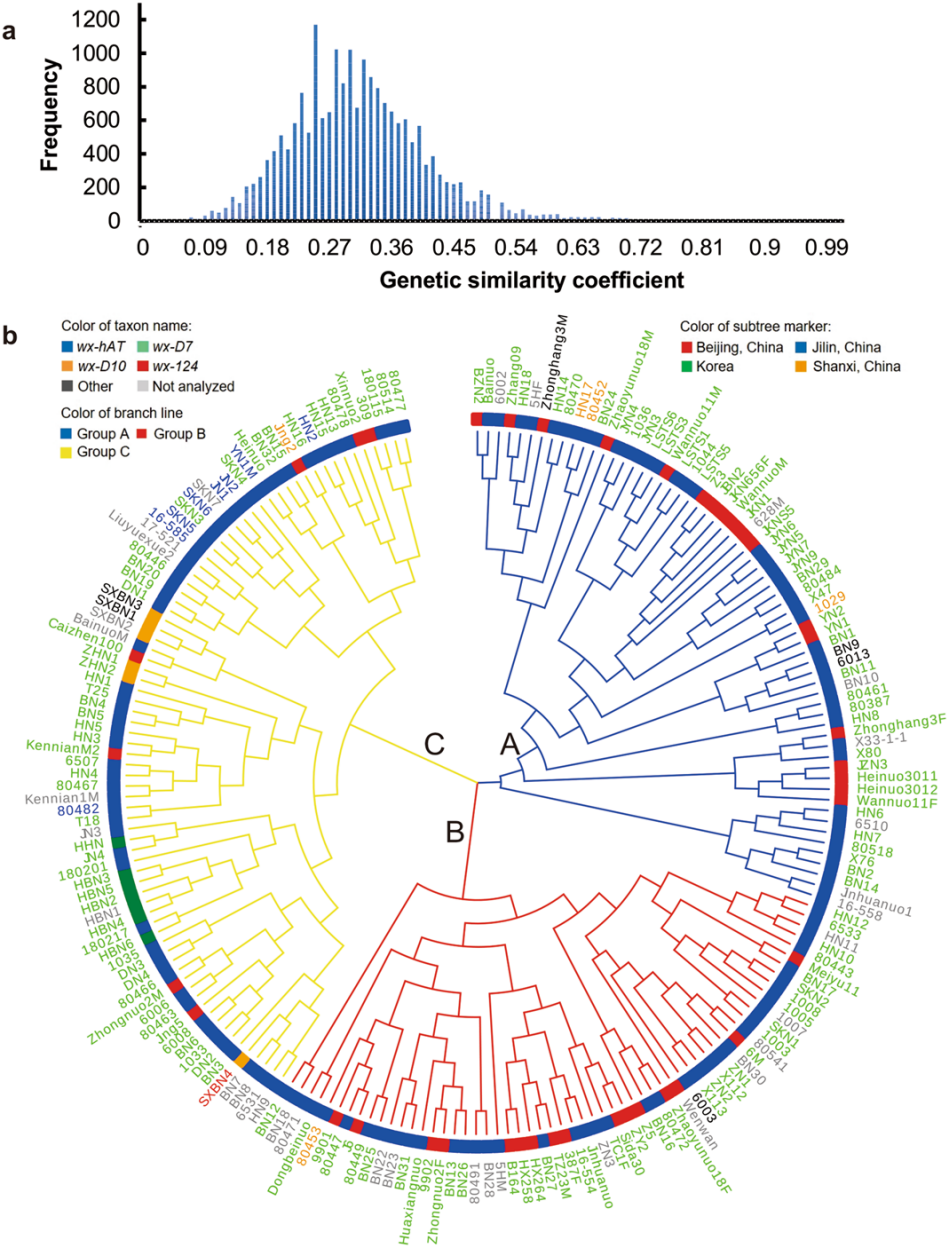
Genetic similarity coefficient and cluster analysis of 200 maize accessions using SSR data. (**a**) Genetic similarity coefficient frequency distribution. The genetic similarity coefficient was calculated by SSRAnalyzer V1.0 (Software copyright registration number: 2018SR003610). (**b**) Cluster analysis of waxy lines. Cluster analysis based on allele identity was carried out using PowerMarker V3.25 with the neighbor-joining method. Different colors of taxon names represent different mutant alleles in *waxy* gene. Different colors of subtree markers represent different origin regions of waxy maize. Different colors of branch lines represent different waxy maize groups. *wx-hAT*: waxy maize with *wx-hAT* mutant allele; *wx-D7*: waxy maize with *wx-D7* mutant allele; *wx-D10*: waxy maize with *wx-D10* mutant allele; *wx-124*: waxy maize with *wx-124* mutant allele; Other: waxy maize had other mutation in *waxy* gene, which was different from *wx-hAT*, *wx-D7*, *wx-D10* and *wx-124*; Not analyzed: the sequence of these waxy maize lines were not obtained; Beijing, China: waxy maize originated from Beijing city in China; Jilin, China: waxy maize originated from Jilin province of China; Shanxi, China: waxy maize originated from Shanxi province of China; Korea: waxy maize originated from Korea; Group A: waxy maize classified into group A; Group B: waxy maize classified into group B; Group C: waxy maize classified into group C.

The genetic diversity of the 200 waxy maize inbred lines (Supplementary Table S2) was evaluated using SSR markers. In total, 458 alleles were found at the 40 SSR loci, with a range of 2 to 25 alleles per marker. The average number of alleles per marker locus across genotypes was 11.45, about double that obtained by Zheng et al.^14^ using 20 SSR markers and 165 accessions. The polymorphism information content (PIC) values for the 40 SSR loci ranged from 0.17 to 0.89 (average, 0.7). The genetic similarity coefficient was analyzed using the SSR data. The genetic similarity coefficient of 200 waxy maize inbred lines ranged from 0.03 to 0.95, with an average of 0.31, and 86.79% of them were less than 0.45 (Fig. 1a). On the basis of similarities of SSR data, a cluster analysis of the 200 waxy maize inbred lines was performed using the neighbor-joining method^18^. The cluster analysis grouped the 200 waxy inbred lines into three main groups: group A included 63 accessions, group B included 59 accessions, and group C included 78 accessions (Fig. 1b). These results show that there was wide genetic diversity among the tested waxy maize accessions.

The genetic distances of waxy maize inbred lines in group A, B and C ranged from 0.11 to 0.97, 0.05 to 0.94, 0.06 to 0.96, with the average values of 0.68, 0.66 and 0.67, respectively. All the three groups contained waxy maize inbred lines originated from Beijing and Jilin province of China, which indicated that waxy maize with different genetic background had been widely used in China, which was consistent with the rapid development of waxy maize breeding and industry in China in recent years. It is worth mentioning that the waxy maize inbred lines originated from Shanxi Province of China and Korea only existed in group C, which indicated that the waxy maize inbred lines from Shanxi Province of China had similar genetic background, and those from Korea had similar genetic background (Fig. 1b). As expected, waxy maize inbred lines with similar pedigree were clustered in the same group. For example, waxy maize inbred lines JYN3, JYN4, JYN5, JYN6, JYN7 and JYN9 all had similar pedigree and were clustered in group A; the waxy maize inbred lines DN1, DN2, DN3 and DN4 had similar pedigree and were clustered in group C (Supplementary Table S2 and Fig. 1b).

### Nucleotide variation at *waxy* locus in waxy maize

Nucleotide sequences from the first to the 14^th^ exon of the *waxy* genes were determined using four pairs of primers (Supplementary Fig. S1 and Table S1). We examined the DNA nucleotide variations in an approximately 3523-bp region of *waxy* loci in 167 waxy and 14 flint maize accessions (Supplementary Data File S1). In this region, the variable nucleotide sites of 169 waxy maize and 14 flint maize were 57 and 87, respectively (Table 1). The genetic variation in *waxy* gene was compared among the three different waxy maize groups and the flint maize. All three waxy maize groups and all waxy maize in three groups showed a minimum level of genetic variation at the *waxy* locus. The estimate of nucleotide diversity for waxy maize accessions in group A, group B, group C and all waxy maize in three groups was 0.00173, 0.00097, 0.00228 and 0.00150, respectively, indicating that the genetic diversity of the *waxy* locus differs among waxy maize populations with different genetic backgrounds. Apparent nucleotide diversity was observed at the *waxy* locus in flint maize (8.4 fold) than in waxy maize. Consistently, the values of *S* (number of polymorphic sites), and *K* (average number of pairwise nucleotide differences) were lower in waxy maize accessions than in non-glutinous maize accessions (Table 1). The significant reduction in diversity at the *waxy* locus in waxy maize suggests that modern waxy maize has experienced a genetic bottleneck during its domestication.

**Table 1:** Summary of nucleotide diversity for *waxy* gene within maize taxa.

Taxon	*N*	Site	*S*	*h*	*Pi*	*K*	*D*	D*	F*	*Rm*
Group A of waxy maize	55	2986	47	14	0.00173	5.165	− 1.72610	− 0.43225	− 1.08725	3
Group B of waxy maize	46	2964	45	7	0.00097	2.871	− 2.49714**	− 2.30817	− 2.82834*	2
Group C of waxy maize	66	2625	42	17	0.00228	5.985	− 1.11350	− 0.40041	− 0.80175	9
Waxy maize in groups A, B and C	167	2582	57	30	0.00150	3.861	− 1.91789*	− 1.95696	− 2.33063*	10
Flint maize	14	3035	87	8	0.01266	38.418	1.72837	1.63115**	1.90528**	6

*N:* number of sequences, Site: number of sites (excluding sites with gaps/missing data), *S*: variable (polymorphic) sites, *h*: number of haplotypes, *Pi*: nucleotide diversity, *K*: average number of pairwise nucleotide differences, *D*: Tajima's D, D*: Fu and Li's D* test, F*: Fu and Li's F* test, *Rm*: Minimum number of recombination events. *P < 0.05, **P < 0.01.

The Tajima’s *D* and Li & Fu’s D* and F* values were calculated to test the deviation from the neutral equilibrium model. All three tests identified negative selection at the *waxy* locus in waxy maize populations, but not in flint maize, suggesting that there has been strong selection acting on waxy maize accessions (Table 1).

The haplotypes of *waxy* gene in waxy maize and flint maize were calculated using DnaSP^19^ software and the results were shown in Table 2. Thirty haplotypes were detected in 167 waxy maize accessions, among which 117 lines shared haplotype Hap_19. Eight haplotypes were detected in 14 flint maize accessions.

**Table 2:** Haplotype of *waxy* gene in studied 167 waxy maize and 14 flint maize.

Haplotype	Variation position	Frequency
**Waxy maize**		
Hap_1	CCAGGAGCTAACCTCGCTGGATAAGGAAAACCAGTGATGCATTCCCGGAAGTATTTG	2
Hap_2	…………TGTCGG…C…………………………….	3
Hap_3	…………………C…………………………….	1
Hap_4	….A.A.G.G…………..A…………………………	1
Hap_5	….A.A.G.G……….C…………………………….	1
Hap_6	.A………….A..A..C……TGA………GCC…CC.CA……	5
Hap_7	.A…T………A..A..C…..GTG…..CTC…C.T.T……….	1
Hap_8	.A…..T…T……A..C……T.A…………………….	1
Hap_9	G.C….T…T………C……T.A.G..CTC…CC.T….CA.G.AG	1
Hap_10	G……T…T………C……T.A.G.ACTC…CC.T….CA.GGAG	1
Hap_11	G……T…T………C……T.A.G..CTCTAGCC.T….CA……	1
Hap_12	G……T…T………C……T.A.G..CTC…CC.T….CAA.GA.	1
Hap_13	G……T…T……..GC……T.A.G..CTC…CC.T….CA……	1
Hap_14	G……T…T…….A.C……T.A.G..CTC…CC.T….CA……	1
Hap_15	G……T…T………CG…G.T.A.G..CTC…CC.T….CA.G…	1
Hap_16	G…A..T…T………C……T.A.G..CTC…CC.T….CA……	2
Hap_17	G…A..TG.GT………C……T.A.G..CTC…CC.T….CA……	2
Hap_18	G…A..TG..T………C……T.A.G..CTC…CC.T….CA……	4
Hap_19	G……T…T………C……T.A.G..CTC…CC.T….CA……	117
Hap_20	G……T…T………C……T.A.G..CTC…CC.T….CA.G…	7
Hap_21	G……T…T………C……T.A.G..CTC..TCC.T….CA……	2
Hap_22	G..A…T…T………C……T.A.G..CTC…CC.T….CA……	1
Hap_23	G……TG..T………C……T.A.G..CTC…CC.T….CA……	1
Hap_24	G……T…T………C……T.A.G..CTC…CC.T….CA…..C	1
Hap_25	G……T…T………C.CA…T.A.G..CTC…CC.T….CA……	1
Hap_26	G……T…T………C……T.A.GA.CTC…CC.T….CA……	1
Hap_27	G……T.G.T………C……T.A.G..CTC…CC.T….CA……	1
Hap_28	G…………..A..A..C……T..G…CTC…C.T.T……….	1
Hap_29	G…….G……A..A..C……T…G..CTC…CC…..GC……	1
Hap_30	G…………..A..A..C……T…G..CTC…CC…..GC……	3
**Flint maize**		
Hap_1	TCGTCCTCCCCGCGTGAGTTCTATGACTTTCGGCCCCCCGTCGCCCCTGAACTGCCTCACTACTACCGACTCACCCCTCCGGGGGCG	1
Hap_2	CTAATTCGGGTA.AGTTCCATACACGTGGCATCATTTTGCA…………..TGTCGG…G.G.CGAT.TTT.C..T..CA.	3
Hap_3	CTAATTCGGGTAGAGTTCCATACACGTGGCATCATTTTGCAGTTGTAACTGAAC….G..GTG.A.A.GATG……..CC.A.A	2
Hap_4	…………………………………………………………GA…….TT..T….AA	3
Hap_5	………………………………………………………..A…………T…..A.A	1
Hap_6	………………………………………………………..A…………………	1
Hap_7	……………………………………..GTAACTGAACT…G..G…A.A.GAT..TT……..AA	2
Hap_8	……………………………………..GT……..T…G..G….GA.GAT..TT……..AA	1

### Phylogenetic analysis based on sequence polymorphisms

**Figure 2:**
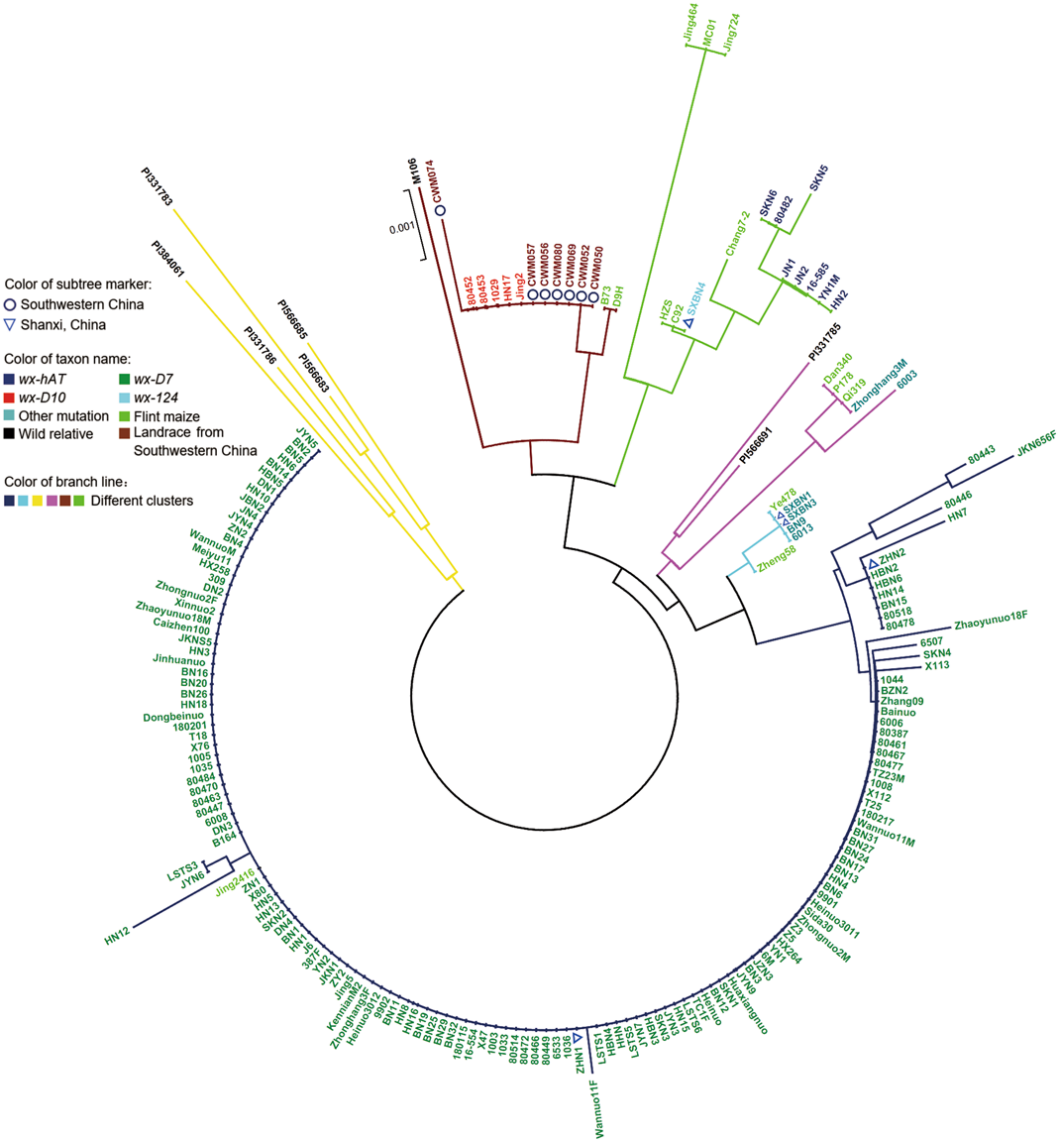
Phylogenetic analysis of maize accessions based on *waxy* gene. The neighbor-joining phylogenetic tree based on the Kimura 2-parameter model was constructed with MEGAX64 software using *waxy* gene sequence data with 1000 bootstrap replicates to assess tree reliability. Different colors of branch lines represent different groups. Different colors of taxon names represent waxy maize inbred lines carrying different *waxy* gene mutation alleles, and flint maize, wild relatives of maize, as well as landraces from Southwestern China. Subtree markers pointed out landraces from Southwestern China and waxy maize inbred lines from Shanxi province of China. *wx-hAT*: waxy maize with *wx-hAT* mutant allele; *wx-D7*: waxy maize with *wx-D7* mutant allele; *wx-D10*: waxy maize with *wx-D10* mutant allele; *wx-124*: waxy maize with *wx-124* mutant allele; Other mutation: waxy maize had other mutation in *waxy* gene, which was different from *wx-hAT*, *wx-D7*, *wx-D10* and *wx-124*; Shanxi, China: waxy maize inbred lines originated from Shanxi province of China; Southwestern China: landraces from Southwestern China.

We obtained the sequences of the 3523-bp region at the *waxy* loci in 167 waxy and 14 flint maize. *Waxy* sequence data for eight wild relatives of maize and seven landraces from Southwestern China were downloaded from the GenBank database. Based on these sequences, a phylogenetic tree including waxy maize inbred lines, flint maize, waxy maize landraces and their relatives was constructed by the neighbor-joining method^20^. According to the tree, five wild relatives of maize formed a branch that was basal to five separate branches. All five branches contained waxy maize and flint maize, indicating that a number of glutinous maize accessions may have been developed from domesticated non-glutinous maize. Qbviously, maize with the same mutation allele clustered together, and waxy maize in different branches carried different mutation alleles. Five waxy maize inbred lines and seven waxy maize landraces formed a branch, and these maize accessions carried the *wx-D10* allele in *waxy* gene. In another branch, eight waxy maize with the *wx-hAT* allele formed a subgroup, and then clustered with one waxy maize harboring the *wx-124* mutant allele. The other two branches contained two and four waxy maize inbred lines, respectively. No insertion or deletion mutations were detected in the amplification region of *waxy* gene in these maize lines, suggesting that they had other mutation alleles in other regions of the *waxy* gene. Furthermore, the remaining 147 waxy inbred lines carrying the *wx-D7* mutant allele clustered together and formed an independent branch, which was significantly separated from waxy maize carrying *wx-D10*, *wx-124* and *wx-hAT* mutant alleles (Fig. 2).

### Identification of accessions with novel *waxy* genotype

**Figure 3:**
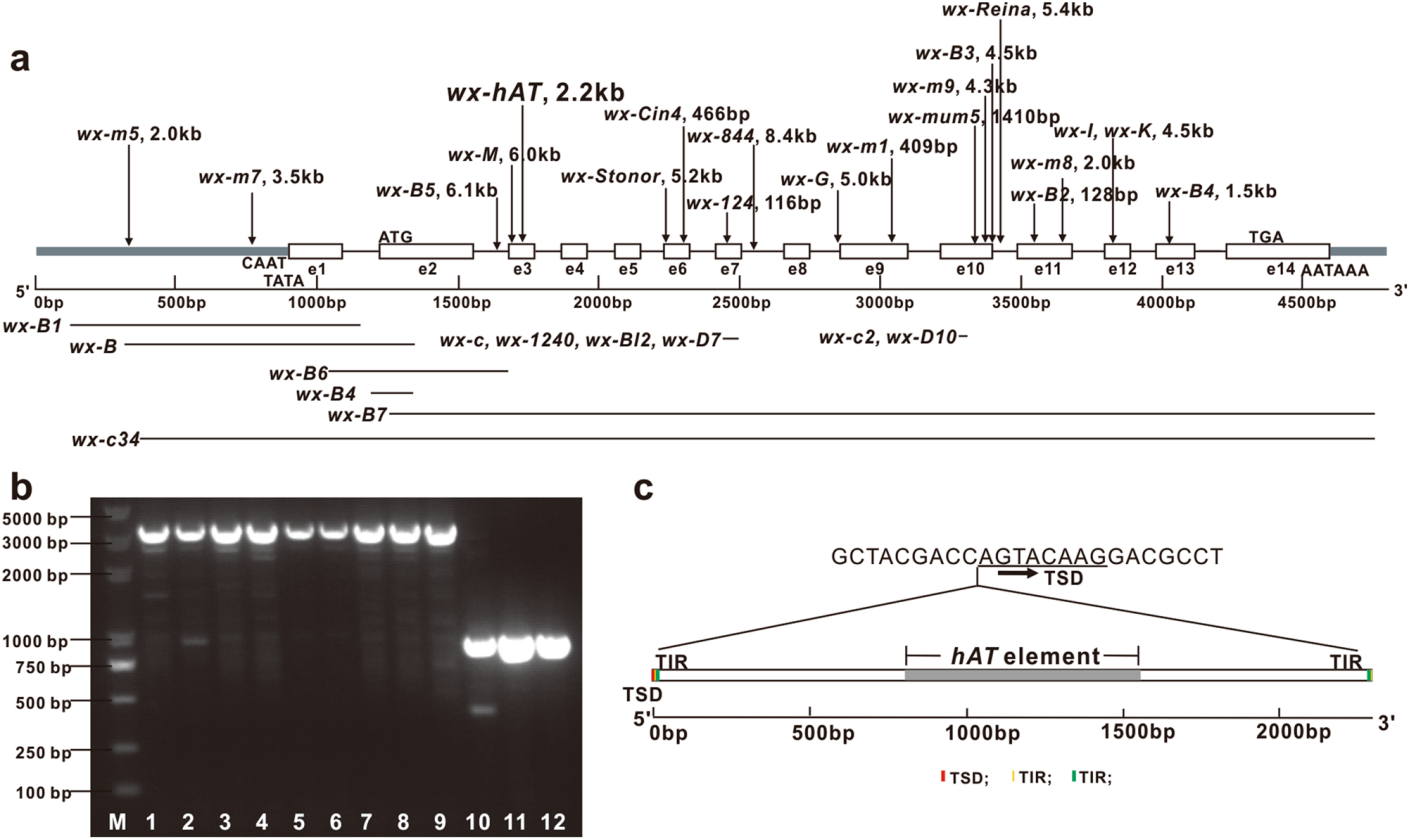
Mutation types of *waxy* gene. (**a**) Wild-type *waxy* gene is about 4.5 kb long and contains 14 exons numbered e1 to e14. Important gene cassettes in promoter region as well as the start and stop codons are indicated. Arrows above schematic of *waxy* gene mark sites of insertion mutations; lines below mark sites of deletion mutations. *wx-hAT* is a new mutation identified in this study. (**b**) Detection of *wx-hAT* mutation by 1% agarose electrophoresis of PCR products. M, marker; lane 1–9, homozygous-type *wx-hAT*; lane 10–12, wild-type *waxy* gene. (**c**) Structural features of *wx-hAT waxy* gene mutation. *wx-hAT* is 2286 bp long, and contains a partial-length of the hAT element (grey box), 8-bp TSD (red box), 3-bp (yellow box) and 9-bp (green box) TIRs.

After sequencing, four types of *waxy* mutation were identified among the waxy maize accessions: 147 accessions carried the *wx-D7* allele with a 30-bp deletion in the seventh exon–intron region; five maize accessions carried the *wx-D10* allele with a 15-bp deletion in the 10^th^ exon region; one maize accession carried the *wx-124* allele with a 125-bp insertion in the seventh exon; and eight maize accessions carried an allele with a new insertion mutation. This mutation was identified as a 2.2 kb insertion in the middle of the third exon of *waxy* gene, which would lead to abnormal gene coding (Fig. 3). The conservative domain of *waxy* gene was analyzed in Pfam database (https://pfam.xfam.org/search/sequence), and found that the 2.2 kb insertion mutation was located on the conserved starch synthase catalytic domain of *waxy* gene. The amylopectin content analysis showed that all eight of these accessions had a high amylopectin content (> 94.5%) in grain starch, like that in *wx-D7* mutant maize lines (Supplementary Table S3). Consistently, the GBSS activity in seeds of these accessions (19.9–28.1 nmol/min/g) was remarkably lower than that in wild type flint maize (66.7–79.3 nmol/min/g) (Supplementary Table S4).

As shown in Fig. 1b, waxy maize in the same group had different origins and carried different *waxy* mutation alleles based on cluster analysis using SSR data. Waxy maize from the same origin possessed different *waxy* mutation alleles. Therefore, based on cluster analysis, there was no correlation among the groups, origins and *waxy* mutation alleles in waxy maize in this study.

### Molecular characteristics of *wx-hAT*

The newly identified insertion mutation was 2286 bp in length and was inserted after the 43^rd^ nucleotide in the third exon of the *waxy* gene (Fig. 3a,b and Supplementary Data File S1). Transposon analysis using the CENSOR program^21^ indicated that the 2.2-kb insertion contained part of the hAT transposon with sequences ranging in length from 813 to 1558 bp (Fig. 3c). We named this *waxy* allele *wx-hAT*. Further analyses revealed that *wx-hAT* had a 5′-CAG-3′ terminal inverted repeat (TIR) and a 5′-GGCGGATCT-3′ near-terminal inverted repeat, generated a 5′-AGTACAAG-3′ target site repeat (TSD). The 3-bp TIR was flanked by the 8-bp TSD and the 3-bp and the 9-bp TIRs were separated by one nucleotide (Fig. 3c). BLAST searches in the MaizeGDB database revealed that the sequences from 9 to 1559 bp and from 1561 to 2286 bp of *wx-hAT* showed 99.03% (e-value = 0) and 99.59% (e-value = 0) identities to the regions of 27,081,986–27,083,536 bp and 27,084,569–27,085,294 bp on chromosome 4 (Supplementary Fig. S2), respectively. This result suggests that this transposon might have jumped from chromosome 4 to the *waxy* gene on chromosome 9. Those regions of chromosome 4 were not annotated.

### Intragenic selection marker for recessive *waxy* gene

**Figure 4:**
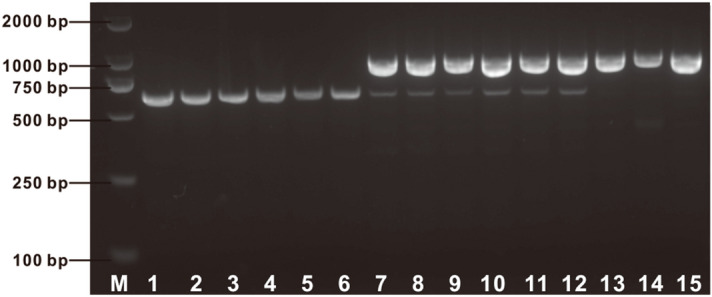
Molecular marker detection of *wx-hAT* mutation by 1.5% agarose electrophoresis of PCR products. M, marker; lane 1–6, SKN5, JN1, HN2, YN1M, 16–585, 80,482, and these waxy maize lines carried the homozygous-type *wx-hAT*; lane 7–12, SKN5 × B73, JN1 × B73, HN2 × B73, YN1M × B73, 16–585 × B73, 80,482 × B73, and these lines carried the heterozygous-type *waxy* gene; lane 13–15, Jing2416, MC01, Jing 464, and these lines carried the wild-type *waxy* gene.

As shown in Fig. 3b, using the primers of E1-4F/E1-4R two amplicons 3.1 kb and 884 bp in length were amplified from waxy lines and flint lines, respectively. We anticipated that the mutation of the *waxy* gene in these waxy lines was caused by the insertion of the 2.2 kb fragment in its allelic dominant gene. However, the heterozygous type and the wild type of the *wx-hAT* allele could not be differentiated by analyzing fragments amplified using this set of PCR primers, because only the 884 bp fragment amplified from the wild-type *waxy* gene was amplified in the heterozygous type maize, which may due to the effect of long fragment amplification being affected by the priority amplification of short fragment. A forward primer waxyF2 was designed on the basis of the 2.2 kb insertion sequence (Supplementary Table S1). Using the three primers E1-4F, E1-4R and waxyF2, a 608-bp and an 884-bp fragment were amplified for the homozygous mutant *waxy* gene and the wild type *waxy* gene, respectively, while both the 608-bp and the 884-bp fragments were amplified for the heterozygous-type *waxy* gene (Fig. 4). Therefore, this set of three primers functioned as a molecular marker for selection of the 2.2-kb insertion mutant of the *waxy* locus. This molecular marker was able to distinguish among maize lines with wild-type, homozygous mutant-type, and heterozygous-type *waxy* genes.

## Discussion

Because of the popularity of glutinous maize in China, waxy maize lines are frequently selected by maize breeders. An abundant of waxy maize germplasm has been obtained through decades of waxy maize breeding. The aim of our study was to reveal the genetic diversity and relationships among modern waxy maize inbred lines, as well as to identify new mutant alleles of the *waxy* gene in maize.

In previous studies, SSR markers have been widely used for genetic analyses of date palm^22^, barley^23^, angelica gigas^24^ and maize^14,25^. In this study, 40 core SSR markers with proven performance for maize genotype identification^26^ were used to genetically analyze 200 waxy maize accessions. The high average PIC value (0.7), high average number of alleles (11.45) and low average genetic similarity coefficient (0.31) are indicative of a high degree of genetic diversity among the waxy maize germplasm.

Compared with rice, maize shows a much higher level of sequence variation at the *waxy* locus^8^. To explore the genetic differentiation of the *waxy* gene in maize, the *waxy* gene sequences (3523 bp) from 167 waxy maize and 14 flint maize were compared and analyzed. The results show that the sequence diversity at the *waxy* locus in waxy maize is only 11.8% of that in flint maize (Table 1). This result is consistent with the findings of previous studies, i.e., that the DNA polymorphism of the *waxy* gene is much reduced in waxy maize^10,13,14^. Consistent the lower level of genetic diversity at the *waxy* locus in waxy maize than in non-glutinous maize, the neutral test revealed negative selection for the *waxy* gene in waxy maize, but not in non-glutinous maize (Table 1). Similar domestication selection in the *waxy* genomic region has also been detected in rice^27–29^, suggesting that the *waxy* gene in waxy crops has experienced a genetic bottleneck and strong artificial selection has acted on this locus during the improvement of waxy crops.

We conducted a phylogenetic analysis using the nucleotide sequences of the *waxy* gene in 167 waxy maize accessions, including five waxy maize accessions (Jing2, HN17, 1029, 80,453, 80,452) with the *wx-D10* allele, one waxy maize accession with the *wx-124* allele (SXBN4), eight waxy maize with the *wx-hAT* allele (SKN6, 80,482, SKN5, JN1, JN2, 16–585, YN1M and HN2), six waxy maize accessions with other *waxy* allele (SXBN1, SXBN3, BN9, 6013, 6003 and Zhonghang3M), and 147 waxy maize accessions with the *wx-D7* allele. A previous study showed that the waxy maize with the *wx-D10* genotype originated from the Yunnan-Guangxi region while that with the *wx-D7* genotype originated from the Yangzi River region^10^. Consistent with these findings, waxy maize harboring *wx-D10* formed a branch while waxy maize with the *wx-D7* mutation was on a distinct independent branch in the tree (Fig. 2). Some waxy maize accessions had *wx-124* and *wx-hAT* mutations and were located on a separate branch, suggesting that there may be additional origins of waxy maize.

The phylogenetic tree showed a clear genetic relationship among waxy maize, flint maize, waxy maize landraces and their wild relatives. Five wild maize relatives (*parviglumis* P1331783, PI1331786 and PI384061, and *mexicana* P1566683 and PI56685) formed a branch that was basal to all flint maize and waxy maize accessions, indicating that wild maize relatives (*Mexicana *and* parviglumis*) might be the ancestor of maize. Seven waxy maize landraces (CWM057, CWM056, CWM069, CWM052, CWM050) and five inbred lines (Jing2, HN17, 1029, 80,453, 80,452) clustered together first and then with one wild relative of maize (*parviglumis* M106) and two flint maize accessions (B73 and D9H) to form an independent branch. All waxy maize in this branch carried the *wx-D10* allele. Next to this branch, eight maize inbred lines (SKN6, 80,482, SKN5, JN1, JN2, 16-585, YN1M and HN2) carrying the *wx-124* allele clustered together first and then with one waxy maize inbred line (SXBN4) harboring the *wx-124* allele, as well as six flint maize (Jing464, MC01, Jing724, HZS, C92 and Chang7-2) to form a separate branch. At the same time, far away from these two branches, two waxy maize (Zhonghang3M and 6003) were intermixed with three flint maize (Dan340, P178 and Qi319), and then clustered with two maize wild relatives (*parviglumis* P1331785 and *mexicana* P1566691) to form an independent branch. Four waxy maize (SXBN1, SXBN3, BN9 and 6013) and two flint maize (Ye478 and Zheng58) formed another branch. The remaining 147 waxy maize carrying the *wx-D7* allele formed a separate branch with one flint maize (Jing2416) (Fig. 2). Waxy maize and flint maize were intermixed in each branch, which indicated that some waxy maize might be domesticated from flint maize. Moreover, waxy maize carrying the *wx-D7* mutation was most abundant in the collected waxy maize inbred lines, suggesting that *wx-D7* might be the main mutation type used in modern waxy maize breeding.

More than 50 waxy maize mutations had been described previously^8^. Previous studies identified two deletion mutations including *wx-D7* and *wx-D10*^10,13^, together with three insertion mutations including *wx-Cin4*^11^, *wx-124*^11^ and *wx-Reina*^3^ in Chinese waxy maize accessions. The *wx-D7*, *wx-D10*, and *wx-124* mutations were also identified in our study, and *wx-D7* was the main waxy mutant allele type among the accessions we studied. Furthermore, we found a new insertion mutation allele, *wx-hAT*, in eight of 200 waxy maize accessions. A truncated hAT element was found in the insertion sequence, so we named this new insertion mutation *wx-hAT*. The hAT element is 3182-bp DNA transposon containing a 14-bp TIR flanked by an 8-bp TSD^30^. Only 746 nucleotides of the hAT element were retained in *wx-hAT* allele. The whole *wx-hAT* sequence was 2286 bp long with a 3-bp TIR flanked by an 8-bp TSD as well as another 9-bp TIR near the terminal (Fig. 3c). The identification of a new allele of the *waxy* gene in this study enriches the collection of maize *waxy* alleles and will be useful for breeding and germplasm preservation.

Interestingly, by comparing sequences with the B73 reference genome, we detected 9–12 bp deletions in the gene sequence of 5′-CAGCACCAGCAGCAG-3′ in the second exon of the *waxy* gene in nine flint maize and all waxy maize accessions (Supplementary Data File S1). Flint maize accessions have this deletion mutation at the *waxy* locus, suggesting that this 15-bp nucleotide sequence is not required for the function of the wild-type *waxy* gene.

To detect *wx-hAT* effectively, we developed PCR molecular markers for this allele. Our results show that the *wx-hAT* mutation can be readily amplified using the primers E1-4Fb, E1-4Rb, and waxyF2. This set of primers amplify an 884-bp product for the wild-type *waxy* gene, a 608-bp product for the mutant type and two fragments (884 bp and 608 bp) for the heterozygous-type. Therefore, these allele-specific primers can be used as molecular markers to discriminate among mutant type, heterozygous-type, and wild type allelic genotypes.

## Materials and methods

### Germplasm accessions, amylopectin content and GBSS activity analysis

The research materials, 200 waxy and 14 flint maize inbred lines, were provided by the Maize Research Center, Beijing Academy of Agriculture and Forestry Sciences (BAAFS). The starch content in seeds was measured according to the National Standards of People’s Republic of China, GB 5009.9-2016. The amylopectin content of seeds was determined using a commercial amylose/amylopectin assay kit (Megazyme, Wicklow, Ireland). The activity of GBSS in harvested maize seeds was determined using a commercial GBSS assay kit (Ziker, Shenzhen, China).

### SSR analyses

Genomic DNA was extracted from leaves using the CTAB procedure^31^. The core 40 SSR markers were developed by BAAFS^26^, and have been approved by the state of China for maize DNA fingerprinting. The PCR protocols and reaction conditions were those specified in the Sector Standard of Agriculture (NY/T 1432-2014). The core 40 SSR primers covered the entire maize genome^26^. The SSR primers were labeled with fluorescent dyes during amplification and the SSR DNA fragments were separated by capillary electrophoresis. The polymorphism information content (PIC) and allele number for each marker was determined using PowerMarker V3.25 software^18^. The genetic similarity coefficient was calculated by SSRAnalyzer V1.0 (Software copyright registration number: 2018SR003610). Cluster analysis based on allele identity was carried out using PowerMarker V3.25 with the neighbor-joining method^18^.

### PCR and DNA sequencing

The *waxy* genes were sequenced from the first to the 14th exon. Primers specific for the *waxy* gene were designed using the Primer3 software (https://primer3.ut.ee/). Exons 1*–*4, exons 4*–*8, exons 8*–*12, and exons 12*–*14 were amplified using the primer pairs 1-4F/R (5′-AGAAGTGTACTGCTCCGTCC-3′ and 5′-AGAACCTGACCGTCTCGTAC-3′), 4-8F/R (5′-TACGAGACGGTCAGGTTC-3′and 5′- GGTAGGAGATGTTGTGGAT-3′)^11^, 8-12F/R (5′-GATTTCATCGACGGGTCTGT-3′and 5′-TCTGTCCCTCTCGTCAGGAT-3′)^14^ and 12-14F/R (5′-ATCCTGACGAGAGGGACAGA-3′ and 5′- CACCGAACAGCAGGGATTAT-3′)^14^, respectively (Supplementary Table S1). Phanta Max Super-Fidelity DNA Polymerase (Vazyme Biotech Nanjing, China) was used for PCR amplification. For PCR with the 1-4F/R primers, a PCR enhancer (Vazyme Biotech, Nanjing, China) was used with the following program: 5 min at 94 °C, 34 cycles of 60 s at 94 °C, 60 s at 59 °C, 2 min at 72 °C, and final extension for 10 min at 72 °C. The PCR enhancer was not added to the PCR reaction mixtures using the other primers. For PCRs with the 4-8F/R primers, the PCR amplification program was as follows: 5 min at 94 °C, 34 cycles of 60 s at 94 °C, 60 s at 55 °C, 2 min at 72 °C, and final extension for 10 min at 72 °C. For PCRs with the of 8-12F/R and 12-14F/R primers, the following program was used: 5 min at 94 °C, 34 cycles of 60 s at 94 °C, 60 s at 60 °C, 2 min at 72 °C, and final extension for 10 min at 72 °C. The PCR products were purified and sequenced by the TsingKe Biological Technology Co. Ltd. (Beijing, China). The gene sequences obtained in this study were shown in Supplementary Data File S1 and were also submitted to the NCBI GenBank database (MT863356–MT863536).

### Sequence alignment analysis

The *waxy* gene sequences of the wild relatives (*Zea mays ssp. Mexicana* and *Zea mays ssp. Parviglumis*) of maize and waxy maize landraces had been studied in previous studies^14^, and they were downloaded from the GenBank database (https://blast.ncbi.nlm.nih.gov/Blast.cgi). Muscle3.8.31_i86win32 software was used for multiple sequence alignment with manual refinement^32^.

### Nucleotide variation analysis

Nucleotide variation analysis was performed with the DnaSP_v61203_x64 program^19^. The number of sites, number of polymorphic sites (*S*), haplotypes (*h*), number of haplotypes, nucleotide diversity (*Pi*), average number of pairwise nucleotide differences (*K*), minimum number of recombination events (*Rm*), and neutrality tests (Tajuma’s D*, Fu and Li's D* and F* test) were calculated using DnaSP software.

### Phylogenetic tree reconstruction

A neighbor-joining phylogenetic tree based on the Kimura 2-parameter model was constructed with MEGAX64 software using *waxy* gene sequence data with 1000 bootstrap replicates to assess tree reliability^20^.

### PCR marker development

The sequence of a newly identified insertion mutant of the *waxy* gene was used to develop a PCR molecular marker. The molecular marker was designed with Primer3 software. The primers were 1-4F (5′-AGAAGTGTACTGCTCCGTCC-3′), 1-4R (5′-AGAACCTGACCGTCTCGTAC-3′), and WaxyF2 (5′-AGTATTGCTTCTACCTGTGGCA-3′) (Supplementary Table S1). The PCR reaction conditions were as follows: 5 min at 94 °C, 34 cycles of 60 s at 94 °C, 60 s at 60 °C, 2 min at 72 °C, and final extension for 10 min at 72 °C. The PCR products were detected by 1.5% agarose gel electrophoresis.

### Sequence analysis

Transposon prediction of the inserted sequence was performed using CENSOR (https://www.girinst.org/censor/index.php)^21^. The similarity between the inserted sequence and the B73 reference sequence was compared by conducting BLAST searches against the MaizeGDB database (https://www.maizegdb.org). Gene structure was drawn by Gene Structure Display Server (https://gsds.cbi.pku.edu.cn/)^33^.

## Supplementary information

Supplementary Information 1.

Supplementary Information 2.
