# Supplementary material for: Sequence polymorphism of the waxy gene in waxy maize accessions and characterization of a new waxy allele
Source: Sci Rep. 2020 Sep 28;10:15851. doi: 10.1038/s41598-020-72764-3 (PMC7522969; doi:10.1038/s41598-020-72764-3)

Sequence polymorphism of the *waxy* gene in waxy maize accessions and characterization of a new *waxy* allele

Meijie Luo[1†]*, Yaxing Shi[1†], Yang Yang[1†], Yanxin Zhao[1], Yunxia Zhang[1], Yamin Shi[1], Mengsi Kong[1], Chunhui Li[1], Zhen Feng[1], Yanli Fan[1], Li Xu[1], Shengli Xi[1], Baishan Lu[1]* and Jiuran Zhao[1]*

[1]Beijing Key Laboratory of Maize DNA Fingerprinting and Molecular Breeding, Maize Research Center, Beijing Academy of Agriculture and Forestry Sciences (BAAFS), Beijing, 100097, China

* Correspondence: mjluo108@163.com; maizezhao@126.com; maizelu@126.com

[†] These authors contributed equally to this work.

Supplementary material

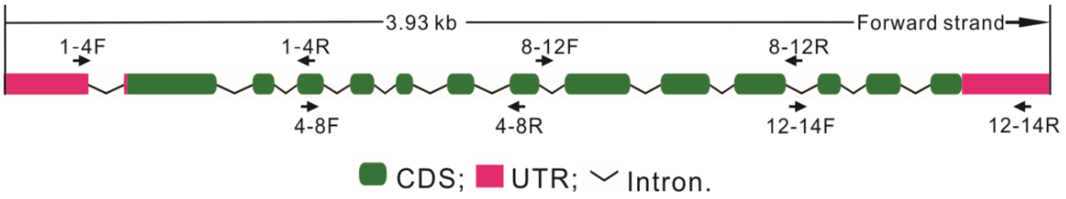

Supplementary Fig. S1 Schematic illustration showing structural features of *waxy* gene (http://www.maizesequence.org/Zea_mays/GRMZM2G024993_T01) and primers used for DNA sequencing in maize

Arrows indicate directions and approximate locations of primers. CDS: coding sequence for amino acids in waxy protein. UTR: untranslated region.

Supplementary Fig. S2 BLAST results of *wx-hAT* sequence against MaizeGDB database

```
Visual alignment for Chr4
Alignment details for Chr4
Alignment details for hit #1 for Chr4
Score =  1503 bits (2776.63),   Expect = 0
Identities = 1536/1551 (0.9903%), Gaps = 6 (0.0039%)
Strand = Plus / Plus


Query  9         CAGGGGCGGATCTACAGGGTATGCCGGGTGGGCCACGGCATACCCTGTAGATTAGGCCCA  68
                 ||||||||||||||||||||||||||||||||||||||||||||||||||||||||||||
Sbjct  27081986  CAGGGGCGGATCTACAGGGTATGCCGGGTGGGCCACGGCATACCCTGTAGATTAGGCCCA  27082045

Query  69        AACTAGCCCATATATGAAGAAAAATAAAAAAAGTCCAGCTTGTATTGGTTCCAGACGTGG  128
                 ||| ||||||||||||||||||||||||||||||||||||||||||||||||||||||||
Sbjct  27082046  AACCAGCCCATATATGAAGAAAAATAAAAAAAGTCCAGCTTGTATTGGTTCCAGACGTGG  27082105

Query  129       GCGATTAGGCGTAGGCTAAGCTGCGAAGCCTTGGGTTCGTCCGGATTTCCGTTCCGTCCG  188
                 ||||| ||||||||||| ||||||||||||| | | |||||| || ||||||||||||||
Sbjct  27082106  GCGAT-AGGCGTAGGCT-AGCTGCGAAGCCT-GCGATCGTCC-GACTTCCGTTCCGTCCG  27082165

Query  189       CTCGTCTTTCTGTTTCTGCCGCCGCCGAGCGCCGTGTCTGTTCATGCTAATCTGACTGCC  248
                 |||||||||||||||||||||||||||||||||||||||| |||||||||||||||||||
Sbjct  27082166  CTCGTCTTTCTGTTTCTGCCGCCGCCGAGCGCCGTGTCTGCTCATGCTAATCTGACTGCC  27082225

Query  249       GCCGACCGCCGCCAAGCGCCAAGTCTGCTCATCTTCATCTACTGCCGGCTGCCTGCCGCC  308
                  |||||||||| ||||||||||||||||||||||||||||||||||||||||||||||||
Sbjct  27082226  ACCGACCGCCG-CAAGCGCCAAGTCTGCTCATCTTCATCTACTGCCGGCTGCCTGCCGCC  27082285

Query  309       GCCGATTGGGCTCGTTGTTTGGTCTTTGGGCTTTGGCAGCCTGTCAGGTACTATACTACC  368
                 ||||||||||||||||||||||||||||||||||||||||||||||||||||||||||||
Sbjct  27082286  GCCGATTGGGCTCGTTGTTTGGTCTTTGGGCTTTGGCAGCCTGTCAGGTACTATACTACC  27082345

Query  369       GTATTATTTGATTACTTATTTCTTTATGACTTTATTGGCTTGCAAATAAATTACGTGATG  428
                 ||||||||||||||||||||||||||||||||||||||||||||||||||||||||||||
Sbjct  27082346  GTATTATTTGATTACTTATTTCTTTATGACTTTATTGGCTTGCAAATAAATTACGTGATG  27082405

Query  429       CCAAAAAAATAGTTTACAATTAGTTTCATGTGTGGTTCGTATATAGCATATGAGTACAGAG  488
                 ||||||||||||||||||||||||||||||||||||||||||||||||||||||||||||
Sbjct  27082406  CCAAAAAAATAGTTTACAATTAGTTTCATGTGTGGTTCGTATATAGCATATGAGTACAGAG  27082465

Query  489       TACTATACGCCTATACTACTAGTACCAAATGATATATATATATATTAGAAACAAATATAATT  548
                 ||||||||||||||||||||||||||||||||||||||||||||||||||||||||||||
Sbjct  27082466  TACTATACGCCTATACTACTAGTACCAAATGATATATATATATATTAGAAACAAATATAATT  27082525

Query  549       GCTACTTTGCTAGTTAGATATTAAAGAACTGTTTGTTGATGCGCTGA-TTTTTTTTACAG  608
                 ||||||||||||||||||||||||||||||||||||||||||||||| ||||||||||||
Sbjct  27082526  GCTACTTTGCTAGTTAGATATTAAAGAACTGTTTGTTGATGCGCTGATTTTTTTTTACAG  27082585

Query  609       TGGCTGAACTATGAAGAGGAATAGCGATATTGCATCACTTTTTCAAAAACATGCAGCAAA  668
                 |||||||||||||||||||||||||||||||||||||||||||||||| |||||||||||
Sbjct  27082586  TGGCTGAACTATGAAGAGGAATAGCGATATTGCATCACTTTTTCAAAACCATGCAGCAAA  27082645

Query  669       GAAGGCTGCTGCTGCTTCATCTCCTTCGCCTGCGGCTCAAGAGAAAGAGACAATAGTGGA  728
                 ||||||||||||||||||||||||||||||||||||||||||||||||||||||||||||
Sbjct  27082646  GAAGGCTGCTGCTGCTTCATCTCCTTCGCCTGCGGCTCAAGAGAAAGAGACAATAGTGGA  27082705

Query  729       AAAAATTATGGATCCTATATCGCCTCCTATTCCTTCGGTTCCACTTGATAATGTCTCACC  788
                 ||||||||||||||||||||||||||||||||||||||||||||||||||||||||||||
Sbjct  27082706  AAAAATTATGGATCCTATATCGCCTCCTATTCCTTCGGTTCCACTTGATAATGTCTCACC  27082765

Query  789       ACCGCCACAATCAGCCCCACCAGTATATGATATTAATCGTCTTCCACAAGATCCAGGTGA  848
                 ||||||||||||||||||||||||||||||||||||||||||||||||||||||||||||
Sbjct  27082766  ACCGCCACAATCAGCCCCACCAGTATATGATATTAATCGTCTTCCACAAGATCCAGGTGA  27082825

Query  849       AAGACAGGCTATTCAAAGCTATGATGTTAACGATCAAGATGCAATTCGAAGATCATATAT  908
                 |||||||||||||||||||||||||||||||||||||||||||||||||||| |||||||
Sbjct  27082826  AAGACAGGCTATTCAAAGCTATGATGTTAACGATCAAGATGCAATTCGAAGAGCATATAT  27082885

Query  909       TCTCAAAGGTCCATTGCAACCATATGCACATGATTTTCCAAAGAGGAAAATTGGGAATAG  968
                 ||||||||||||||||||||||||||||||||||||||||||||||||||||||||||||
Sbjct  27082886  TCTCAAAGGTCCATTGCAACCATATGCACATGATTTTCCAAAGAGGAAAATTGGGAATAG  27082945

Query  969       AGATCGTCAATTCAATTTTGTATGGTTTGCAAATCATCATTGGCTTGAATATAGTATTAA  1028
                 ||||||||||||||||||||||||||||||||||||||||||||||||||||||||||||
Sbjct  27082946  AGATCGTCAATTCAATTTTGTATGGTTTGCAAATCATCATTGGCTTGAATATAGTATTAA  27083005

Query  1029      AAAGGATGACGTGTTTTGCTTTGTATGCTACTTGTTCAGAGATAACAAATGTAAGGGCAA  1088
                 |||||||| |||||||||||||||||||||||||||||||||||||||||||||||||||
Sbjct  27083006  AAAGGATGCCGTGTTTTGCTTTGTATGCTACTTGTTCAGAGATAACAAATGTAAGGGCAA  27083065

Query  1089      GGGTGCAGATACATTCATTACAGATGGTTGGAGAAATTGGAATATAGGAAATAAAGCCTT  1148
                 ||||||||||||||||||||||||||||||||||||||||||||||||||||||||||||
Sbjct  27083066  GGGTGCAGATACATTCATTACAGATGGTTGGAGAAATTGGAATATAGGAAATAAAGCCTT  27083125

Query  1149      ACTGAAACATGTGGGTTCTAGTGCACACAAAGCAGCTCAAGAGAGATACAATGGCTTTGT  1208
                 ||||||||||||||||||||||||||||||||||||||||||||||||||||||||||||
Sbjct  27083126  ACTGAAACATGTGGGTTCTAGTGCACACAAAGCAGCTCAAGAGAGATACAATGGCTTTGT  27083185

Query  1209      GAATCCTACTGCAGCAATTGATTATCACATTGAGAAATGGAGTGATGAGGATCTTCGTCT  1268
                 ||||||||||||||||||||||||||||||||||||||||||||||||||||||||||||
Sbjct  27083186  GAATCCTACTGCAGCAATTGATTATCACATTGAGAAATGGAGTGATGAGGATCTTCGTCT  27083245

Query  1269      TTATAAGATCAGGTTGACTTATTCACTTAAATGTTTAAAGTTTCTTTTGCATCAAGGATT  1328
                 ||||||||||||||||||||||||||||||||||||||||||||||||||||||||||||
Sbjct  27083246  TTATAAGATCAGGTTGACTTATTCACTTAAATGTTTAAAGTTTCTTTTGCATCAAGGATT  27083305

Query  1329      GGCATTTCGTGGACATGATGAAAGTGAAGAGTCTAGCAACAGAGGTAACTTCCTTGAGCT  1388
                 ||||||||||||||||||||||||||||||||||||||||||||||||||||||||||||
Sbjct  27083306  GGCATTTCGTGGACATGATGAAAGTGAAGAGTCTAGCAACAGAGGTAACTTCCTTGAGCT  27083365

Query  1389      TTTGAAATTTCTTGCAGCAAATAGTGAAGAAGTGAATAAGTATGTTTTGAAGAATGCTCC  1448
                 ||||||||||||||||||||||||||||||||||||||||||||||||||||||||||||
Sbjct  27083366  TTTGAAATTTCTTGCAGCAAATAGTGAAGAAGTGAATAAGTATGTTTTGAAGAATGCTCC  27083425

Query  1449      AGGTAATTGCACCCTAACTTGCCCGGATATACAAAAGCAAATTATTCAATGTTGTGCCAT  1508
                 ||||||||||||||||||||||||||||||||||||||||||||||||||||||||||||
Sbjct  27083426  AGGTAATTGCACCCTAACTTGCCCGGATATACAAAAGCAAATTATTCAATGTTGTGCCAT  27083485

Query  1509      AGAAACTAGAAAAAAAAATTATTGAAGAACTTGGTGATGAGCACTATGCAAT  1559
                 |||||||||||||||||||||||||||||||||||||||||||||||||||
Sbjct  27083486  AGAAACTAGAAAAAAAAATTATTGAAGAACTTGGTGATGAGCACTATGCAAT  27083536
```

Alignment details for hit #2 for Chr4

```
Score =  717 bits (1325.17),  Expect = 0
Identities = 723/726 (0.9959%), Gaps = 0 (0.0000%)
Strand = Plus / Plus


Query  1561      AGTTACTTTCTTGTATGTCAGCCTTCAACCCGATCAATTCATTTGCTTCCTTTGATGCCC  1620
                 ||||||||||||||||||||||||||||||||||||||||||||||||||||||||||||
Sbjct  27084569  AGTTACTTTCTTGTATGTCAGCCTTCAACCCGATCAATTCATTTGCTTCCTTTGATGCCC  27084628

Query  1621      AAAAGATACGTAGGCTTGCTGAGTTCTATCCTAATGACATCATCTCAAATGCCAATTTGC  1680
                 ||||||||||||||||||||||||||||||||||||||||||||||||||||||||||||
Sbjct  27084629  AAAAGATACGTAGGCTTGCTGAGTTCTATCCTAATGACATCATCTCAAATGCCAATTTGC  27084688

Query  1681      AAAAGCTTGAGTTGCAACTTGATAACTATATTGATGACATAAGACAAGATGATAGCTTCA  1740
                 ||||||||||||||||||||||||||||||||||||||||||||||||||||||||||||
Sbjct  27084689  AAAAGCTTGAGTTGCAACTTGATAACTATATTGATGACATAAGACAAGATGATAGCTTCA  27084748

Query  1741      AAAGCCTAGGGAATCTTGTTGATCTCTCAGTTAAGCTCGTTGAAACAAAGAGACATATAG  1800
                 ||||||||||||||||||||||||||||||||||||||||||||||||||||||||||||
Sbjct  27084749  AAAGCCTAGGGAATCTTGTTGATCTCTCAGTTAAGCTCGTTGAAACAAAGAGACATATAG  27084808

Query  1801      TTTATGATTTGGTTTACGTGCTTCTCAAATTAGTATTGCTTCTACCTGTGGCAACAGCTA  1860
                 ||||||||||||||||| ||||||||||||||||||||||||||||||||||||||||||
Sbjct  27084809  TTTATGATTTGGTTTACATGCTTCTCAAATTAGTATTGCTTCTACCTGTGGCAACAGCTA  27084868

Query  1861      GTGTTGAGAGGGTATTTTCTGCAATGAGTTTAGTCAAAAGTAAGTTAAGAAATAAGATGG  1920
                 ||||||||||||||||||||||||||||||||||||||||||||||||||||||||||| 
Sbjct  27084869  GTGTTGAGAGGGTATTTTCTGCAATGAGTTTAGTCAAAAGTAAGTTAAGAAATAAGATGA  27084928

Query  1921      GTGATAGTCTTTTGGATGATTGTCTTGTCACGTTCATTGAGAGAGATATTTTTTTCAAGA  1980
                 |||||||||||||||||||||||||||||||||||||||| |||||||||||||||||||
Sbjct  27084929  GTGATAGTCTTTTGGATGATTGTCTTGTCACGTTCATTGAAAGAGATATTTTTTTCAAGA  27084988

Query  1981      TTGATGAAGATGATATAATCAAGAATTTTATGGCCATTAGAAGGCGCCCGACCAAACAAGA  2040
                 ||||||||||||||||||||||||||||||||||||||||||||||||||||||||||||
Sbjct  27084989  TTGATGAAGATGATATAATCAAGAATTTTATGGCCATTAGAAGGCGCCCGACCAAACAAGA  27085048

Query  2041      AGGATAAGAAGTGATTATATATTGATCTACTACAGGTGTGCAATCTTATTTTATGCACGT  2100
                 ||||||||||||||||||||||||||||||||||||||||||||||||||||||||||||
Sbjct  27085049  AGGATAAGAAGTGATTATATATTGATCTACTACAGGTGTGCAATCTTATTTTATGCACGT  27085108

Query  2101      TTTAGATTAAATATGTTCAAACTCCACTTATATTGTAATTTGTGCTGGTTTCTAGCTATT  2160
                 ||||||||||||||||||||||||||||||||||||||||||||||||||||||||||||
Sbjct  27085109  TTTAGATTAAATATGTTCAAACTCCACTTATATTGTAATTTGTGCTGGTTTCTAGCTATT  27085168

Query  2161      TTATATATGTGTACCGAATTGATTGGCAATTTGAAGTTATCTACCGATTTTTTATCTGTT  2220
                 ||||||||||||||||||||||||||||||||||||||||||||||||||||||||||||
Sbjct  27085169  TTATATATGTGTACCGAATTGATTGGCAATTTGAAGTTATCTACCGATTTTTTATCTGTT  27085228

Query  2221      TTTTTAAATTGTATATGAAAATATTAGCTCGGCATACTCTAACTTAAAATCCTAGATCCG  2280
                 ||||||||||||||||||||||||||||||||||||||||||||||||||||||||||||
Sbjct  27085229  TTTTTAAATTGTATATGAAAATATTAGCTCGGCATACTCTAACTTAAAATCCTAGATCCG  27085288

Query  2281      CCACTG  2286
                 ||||||
Sbjct  27085289  CCACTG  27085294
```

Supplementary Table S1 Primers used for PCR

Primer name	F/R	5′–3′ Primers
1-4	F	AGAAGTGTACTGCTCCGTCC
	R	AGAACCTGACCGTCTCGTAC
4-8	F	TACGAGACGGTCAGGTTC
	R	GGTAGGAGATGTTGTGGAT
8-12	F	GATTTCATCGACGGGTCTGT
	R	TCTGTCCCTCTCGTCAGGAT
12-14	F	ATCCTGACGAGAGGGACAGA
	R	CACCGAACAGCAGGGATTAT
WaxyF2	F	AGTATTGCTTCTACCTGTGGCA

Supplementary Table S2 Summary of sampled accessions for genetic variation analysis

Taxon	Accession name	Type	Origin	Seed source or reference	*wx* genotype	Pedigree information
Waxy maize	SKN1	Inbred line	Jilin,China	BAAFS	*wx-7*	unknown
	SKN2	Inbred line	Jilin,China	BAAFS	*wx-7*	unknown
	SKN3	Inbred line	Jilin,China	BAAFS	*wx-7*	unknown
	SKN4	Inbred line	Jilin,China	BAAFS	*wx-7*	unknown
	SKN5	Inbred line	Jilin,China	BAAFS	*wx-hAT*	unknown
	SKN6	Inbred line	Jilin,China	BAAFS	*wx-hAT*	unknown
	SKN7	Inbred line	Jilin,China	BAAFS	Not analyzed	unknown
	JN1	Inbred line	Jilin,China	BAAFS	*wx-hAT*	unknown
	JN2	Inbred line	Jilin,China	BAAFS	*wx-hAT*	unknown
	JN3	Inbred line	Jilin,China	BAAFS	Not analyzed	unknown
	JN4	Inbred line	Jilin,China	BAAFS	*wx-7*	unknown
	Huaxiangnuo	Inbred line	Jilin,China	BAAFS	*wx-7*	unknown
	DN1	Inbred line	Jilin,China	BAAFS	*wx-7*	Selected from Huaxiangnuo
	DN2	Inbred line	Jilin,China	BAAFS	*wx-7*	Selected from Huaxiangnuo
	DN3	Inbred line	Jilin,China	BAAFS	*wx-7*	Selected from Huaxiangnuo
	DN4	Inbred line	Jilin,China	BAAFS	*wx-7*	Selected from Huaxiangnuo
	JYN3	Inbred line	Jilin,China	BAAFS	*wx-7*	Selected from Jinyinnuo
	JYN4	Inbred line	Jilin,China	BAAFS	*wx-7*	Selected from Jinyinnuo
	JYN6	Inbred line	Jilin,China	BAAFS	*wx-7*	Selected from Jinyinnuo
	JYN9	Inbred line	Jilin,China	BAAFS	*wx-7*	Selected from Jinyinnuo
	LSTS1	Inbred line	Jilin,China	BAAFS	*wx-7*	Selecte from Lvsetianshi
	LSTS3	Inbred line	Jilin,China	BAAFS	*wx-7*	Selecte from Lvsetianshi
	LSTS5	Inbred line	Jilin,China	BAAFS	*wx-7*	Selecte from Lvsetianshi
	LSTS6	Inbred line	Jilin,China	BAAFS	*wx-7*	Selecte from Lvsetianshi
	Bainuo	Inbred line	Jilin,China	BAAFS	*wx-7*	unkown
	BN1	Inbred line	Jilin,China	BAAFS	*wx-7*	unkown
	BN2	Inbred line	Jilin,China	BAAFS	*wx-7*	unkown
	ZN1	Inbred line	Jilin,China	BAAFS	*wx-7*	unkown
	ZN2	Inbred line	Jilin,China	BAAFS	*wx-7*	unkown
	BN3	Inbred line	Jilin,China	BAAFS	*wx-7*	unkown
	HN1	Inbred line	Jilin,China	BAAFS	*wx-7*	unkown
	BN4	Inbred line	Jilin,China	BAAFS	*wx-7*	unkown
	BN5	Inbred line	Jilin,China	BAAFS	*wx-7*	unkown
	SXBN1	Inbred line	Shanxi, China	BAAFS	Other	unkown
	SXBN2	Inbred line	Shanxi, China	BAAFS	Not analyzed	unkown
	SXBN3	Inbred line	Shanxi, China	BAAFS	Other	unkown
	ZHN1	Inbred line	Shanxi, China	BAAFS	*wx-7*	unkown
	ZHN2	Inbred line	Shanxi, China	BAAFS	*wx-7*	unkown
	SXBN4	Inbred line	Shanxi, China	BAAFS	*wx-124*	unkown
	HHN	Inbred line	Korea	BAAFS	*wx-7*	unkown
	HBN1	Inbred line	Korea	BAAFS	Not analyzed	unkown
	HBN2	Inbred line	Korea	BAAFS	*wx-7*	unkown

HBN3	Inbred line	Korea	BAAFS	*wx-7*	unkown
HBN4	Inbred line	Korea	BAAFS	*wx-7*	unkown
HBN5	Inbred line	Korea	BAAFS	*wx-7*	unkown
HBN6	Inbred line	Korea	BAAFS	*wx-7*	unkown
BN6	Inbred line	Jilin, China	BAAFS	*wx-7*	unkown
BN7	Inbred line	Jilin, China	BAAFS	Not analyzed	unkown
BN8	Inbred line	Jilin, China	BAAFS	Not analyzed	unkown
BN9	Inbred line	Jilin, China	BAAFS	Other	unkown
BN10	Inbred line	Jilin, China	BAAFS	Not analyzed	unkown
BN11	Inbred line	Jilin, China	BAAFS	*wx-7*	unkown
HN2	Inbred line	Jilin, China	BAAFS	*wx-hAT*	unkown
HN3	Inbred line	Jilin, China	BAAFS	*wx-7*	unkown
HN4	Inbred line	Jilin, China	BAAFS	*wx-7*	unkown
HN5	Inbred line	Jilin, China	BAAFS	*wx-7*	unkown
HN6	Inbred line	Jilin, China	BAAFS	*wx-7*	unkown
HN7	Inbred line	Jilin, China	BAAFS	*wx-7*	unkown
HN8	Inbred line	Jilin, China	BAAFS	*wx-7*	unkown
HN9	Inbred line	Jilin, China	BAAFS	Not analyzed	unkown
HN10	Inbred line	Jilin, China	BAAFS	*wx-7*	unkown
HN11	Inbred line	Jilin, China	BAAFS	Not analyzed	unkown
HN12	Inbred line	Jilin, China	BAAFS	*wx-7*	unkown
BN12	Inbred line	Jilin, China	BAAFS	*wx-7*	unkown
Jinhuanuo	Inbred line	Jilin, China	BAAFS	*wx-7*	Selected from Jinhuanuo
TC1F	Inbred line	Jilin, China	BAAFS	*wx-7*	unkown
YN1M	Inbred line	Jilin, China	BAAFS	*wx-hAT*	unkown
Jinhuanuo1	Inbred line	Jilin, China	BAAFS	Not analyzed	unkown
Liuyuexue2	Inbred line	Jilin, China	BAAFS	Not analyzed	unkown
Kennian1M	Inbred line	Jilin, China	BAAFS	Not analyzed	232-2×Yuanfu17
5HM	Inbred line	Jilin, China	BAAFS	Not analyzed	Selected from Nuo9
5HF	Inbred line	Jilin, China	BAAFS	Not analyzed	Selected from Nuo10
HN13	Inbred line	Jilin, China	BAAFS	*wx-7*	unkown
Heinuo	Inbred line	Jilin, China	BAAFS	*wx-7*	unkown
BN13	Inbred line	Jilin, China	BAAFS	*wx-7*	unkown
BN14	Inbred line	Jilin, China	BAAFS	*wx-7*	unkown
HN14	Inbred line	Jilin, China	BAAFS	*wx-7*	unkown
BN15	Inbred line	Jilin, China	BAAFS	*wx-7*	unkown
HN15	Inbred line	Jilin, China	BAAFS	*wx-7*	unkown
HN16	Inbred line	Jilin, China	BAAFS	*wx-7*	unkown
BN16	Inbred line	Jilin, China	BAAFS	*wx-7*	unkown
BN17	Inbred line	Jilin, China	BAAFS	*wx-7*	unkown
HN17	Inbred line	Jilin, China	BAAFS	*wx-10*	unkown
BN18	Inbred line	Jilin, China	BAAFS	Not analyzed	unkown
BN19	Inbred line	Jilin, China	BAAFS	*wx-7*	unkown
BN20	Inbred line	Jilin, China	BAAFS	*wx-7*	unkown
BN22	Inbred line	Jilin, China	BAAFS	Not analyzed	unkown
BN23	Inbred line	Jilin, China	BAAFS	Not analyzed	unkown
BN24	Inbred line	Jilin, China	BAAFS	*wx-7*	unkown
BN25	Inbred line	Jilin, China	BAAFS	*wx-7*	unkown

BN26	Inbred line	Jilin, China	BAAFS	*wx-7*	unkown
BN27	Inbred line	Jilin, China	BAAFS	*wx-7*	unkown
BN28	Inbred line	Jilin, China	BAAFS	Not analyzed	unkown
BN29	Inbred line	Jilin, China	BAAFS	*wx-7*	unkown
BN30	Inbred line	Jilin, China	BAAFS	Not analyzed	unkown
HN18	Inbred line	Jilin, China	BAAFS	*wx-7*	unkown
BN31	Inbred line	Jilin, China	BAAFS	*wx-7*	unkown
ZN3	Inbred line	Jilin, China	BAAFS	Not analyzed	unkown
BN32	Inbred line	Jilin, China	BAAFS	*wx-7*	unkown
Dongbeinuo	Inbred line	Jilin, China	BAAFS	*wx-7*	unkown
Wannuo11M	Inbred line	Beijing, China	BAAFS	*wx-7*	unkown
JZN3	Inbred line	Beijing, China	BAAFS	*wx-7*	unkown
JBN2	Inbred line	Beijing, China	BAAFS	*wx-7*	Selected from Zinuo3
J6	Inbred line	Beijing, China	BAAFS	*wx-7*	Selected from Zhongnuo1
WannuoM	Inbred line	Beijing, China	BAAFS	*wx-7*	unkown
JKN656F	Inbred line	Beijing, China	BAAFS	*wx-7*	Selected from Jingnuo31
6M	Inbred line	Beijing, China	BAAFS	*wx-7*	unkown
387F	Inbred line	Beijing, China	BAAFS	*wx-7*	unkown
Meiyu11	Inbred line	Beijing, China	BAAFS	*wx-7*	Selected from Meiyu11
YN1	Inbred line	Beijing, China	BAAFS	*wx-7*	unkown
YN2	Inbred line	Beijing, China	BAAFS	*wx-7*	unkown
HX258	Inbred line	Beijing, China	BAAFS	*wx-7*	unkown
HX264	Inbred line	Beijing, China	BAAFS	*wx-7*	unkown
JKN1	Inbred line	Beijing, China	BAAFS	*wx-7*	Selected from Jingkenuo2000
B164	Inbred line	Beijing, China	BAAFS	*wx-7*	unkown
309	Inbred line	Beijing, China	BAAFS	*wx-7*	unkown
Z5	Inbred line	Beijing, China	BAAFS	*wx-7*	unkown
ZY2	Inbred line	Beijing, China	BAAFS	*wx-7*	Selected from Zixiangnuo1
Z3	Inbred line	Beijing, China	BAAFS	*wx-7*	unkown
Jing5	Inbred line	Beijing, China	BAAFS	*wx-7*	unkown
Zhang09	Inbred line	Beijing, China	BAAFS	*wx-7*	unkown
Jing2	Inbred line	Beijing, China	BAAFS	*wx-10*	unkown
Zhongnuo2F	Inbred line	Beijing, China	BAAFS	*wx-7*	Selected from Kennian1
Zhongnuo2M	Inbred line	Beijing, China	BAAFS	*wx-7*	Selected from CTW3446
Wenwan	Inbred line	Beijing, China	BAAFS	Not analyzed	unkown
Wannuo11F	Inbred line	Beijing, China	BAAFS	*wx-7*	unkown
KennianM2	Inbred line	Beijing, China	BAAFS	*wx-7*	Selected from Nuo3
Xinnuo2	Inbred line	Beijing, China	BAAFS	*wx-7*	unkown
Sida30	Inbred line	Beijing, China	BAAFS	*wx-7*	Selected from Sida30
Zhonghang3M	Inbred line	Beijing, China	BAAFS	Other	unkown
Zhonghang3F	Inbred line	Beijing, China	BAAFS	*wx-7*	unkown
Zhaoyunuo18F	Inbred line	Beijing, China	BAAFS	*wx-7*	unkown
Zhaoyunuo18M	Inbred line	Beijing, China	BAAFS	*wx-7*	unkown
Heinuo3011	Inbred line	Beijing, China	BAAFS	*wx-7*	unkown
Heinuo3012	Inbred line	Beijing, China	BAAFS	*wx-7*	unkown
Caizhen100	Inbred line	Beijing, China	BAAFS	*wx-7*	unkown
628M	Inbred line	Beijing, China	BAAFS	Not analyzed	unkown
9901	Inbred line	Beijing, China	BAAFS	*wx-7*	unkown

9902	Inbred line	Beijing, China	BAAFS	*wx-7*	unkown
JKNS5	Inbred line	Beijing, China	BAAFS	*wx-7*	Selected from Jingkenuo2000
80541	Inbred line	Jilin, China	BAAFS	Not analyzed	unkown
180115	Inbred line	Jilin, China	BAAFS	*wx-7*	unkown
180201	Inbred line	Jilin, China	BAAFS	*wx-7*	unkown
180217	Inbred line	Jilin, China	BAAFS	*wx-7*	unkown
16-554	Inbred line	Jilin, China	BAAFS	*wx-7*	unkown
16-558	Inbred line	Jilin, China	BAAFS	Not analyzed	unkown
16-585	Inbred line	Jilin, China	BAAFS	*wx-hAT*	unkown
17-521	Inbred line	Jilin, China	BAAFS	Not analyzed	unkown
T18	Inbred line	Jilin, China	BAAFS	*wx-7*	unkown
T25	Inbred line	Jilin, China	BAAFS	*wx-7*	unkown
X33-1-1	Inbred line	Jilin, China	BAAFS	Not analyzed	unkown
X47	Inbred line	Jilin, China	BAAFS	*wx-7*	unkown
X76	Inbred line	Jilin, China	BAAFS	*wx-7*	unkown
X80	Inbred line	Jilin, China	BAAFS	*wx-7*	unkown
X112	Inbred line	Jilin, China	BAAFS	*wx-7*	unkown
X113	Inbred line	Jilin, China	BAAFS	*wx-7*	unkown
BainuoM	Inbred line	Jilin, China	BAAFS	Not analyzed	unkown
1003	Inbred line	Jilin, China	BAAFS	*wx-7*	unkown
1005	Inbred line	Jilin, China	BAAFS	*wx-7*	unkown
1007	Inbred line	Jilin, China	BAAFS	Not analyzed	unkown
1008	Inbred line	Jilin, China	BAAFS	*wx-7*	unkown
1029	Inbred line	Jilin, China	BAAFS	*wx-10*	unkown
1033	Inbred line	Jilin, China	BAAFS	*wx-7*	unkown
1035	Inbred line	Jilin, China	BAAFS	*wx-7*	unkown
1036	Inbred line	Jilin, China	BAAFS	*wx-7*	unkown
1044	Inbred line	Jilin, China	BAAFS	*wx-7*	unkown
6002	Inbred line	Jilin, China	BAAFS	Not analyzed	unkown
6003	Inbred line	Jilin, China	BAAFS	*other*	unkown
6006	Inbred line	Jilin, China	BAAFS	*wx-7*	unkown
6008	Inbred line	Jilin, China	BAAFS	*wx-7*	unkown
6013	Inbred line	Jilin, China	BAAFS	*other*	unkown
6507	Inbred line	Jilin, China	BAAFS	*wx-7*	unkown
6510	Inbred line	Jilin, China	BAAFS	Not analyzed	unkown
6531	Inbred line	Jilin, China	BAAFS	Not analyzed	unkown
6533	Inbred line	Jilin, China	BAAFS	*wx-7*	unkown
80387	Inbred line	Jilin, China	BAAFS	*wx-7*	unkown
80443	Inbred line	Jilin, China	BAAFS	*wx-7*	unkown
80446	Inbred line	Jilin, China	BAAFS	*wx-7*	unkown
80447	Inbred line	Jilin, China	BAAFS	*wx-7*	unkown
80449	Inbred line	Jilin, China	BAAFS	*wx-7*	unkown
80452	Inbred line	Jilin, China	BAAFS	*wx-10*	unkown
80453	Inbred line	Jilin, China	BAAFS	*wx-10*	unkown
80461	Inbred line	Jilin, China	BAAFS	*wx-7*	unkown
80463	Inbred line	Jilin, China	BAAFS	*wx-7*	unkown
80466	Inbred line	Jilin, China	BAAFS	*wx-7*	unkown
80467	Inbred line	Jilin, China	BAAFS	*wx-7*	unkown

	80470	Inbred line	Jilin, China	BAAFS	*wx-7*	unkown
	80471	Inbred line	Jilin, China	BAAFS	Not analyzed	unkown
	80472	Inbred line	Jilin, China	BAAFS	*wx-7*	unkown
	80477	Inbred line	Jilin, China	BAAFS	*wx-7*	unkown
	80478	Inbred line	Jilin, China	BAAFS	*wx-7*	unkown
	80482	Inbred line	Jilin, China	BAAFS	*wx-hAT*	unkown
	80484	Inbred line	Jilin, China	BAAFS	*wx-7*	unkown
	80491	Inbred line	Jilin, China	BAAFS	Not analyzed	unkown
	80514	Inbred line	Jilin, China	BAAFS	*wx-7*	unkown
	80518	Inbred line	Jilin, China	BAAFS	*wx-7*	unkown
	BZN2	Inbred line	Beijing, China	BAAFS	*wx-7*	unkown
	TZ23M	Inbred line	Beijing, China	BAAFS	*wx-7*	unkown
	JYN5	Inbred line	Jilin, China	BAAFS	*wx-7*	Selected from Jinyinnuo
	JYN7	Inbred line	Jilin, China	BAAFS	*wx-7*	Selected from Jinyinnuo
	CWM050	Landrace	Guizhou, China	Zheng et al., 2013	*wx-10*	unkown
	CWM052	Landrace	Guizhou, China	Zheng et al., 2013	*wx-10*	unkown
	CWM069	Landrace	Yunnan, China	Zheng et al., 2013	*wx-10*	unkown
	CWM056	Landrace	Yunnan, China	Zheng et al., 2013	*wx-10*	unkown
	CWM057	Landrace	Yunnan, China	Zheng et al., 2013	*wx-10*	unkown
	CWM074	Landrace	Yunnan, China	Zheng et al., 2013	*wx-10*	unkown
	CWM080	Landrace	Shanxi, China	Zheng et al., 2013	*wx-10*	unkown
Flint maize	Jing2416	Inbred line	Beijing, China	BAAFS	wild type	Jing24×5237
	Jing464	Inbred line	Beijing, China	BAAFS	wild type	Selected from X1132
	MC01	Inbred line	Beijing, China	BAAFS	wild type	Selected from X1132
	HZS	Inbred line	Beijing, China	BAAFS	wild type	Selected from Tangsipingtou
	Chang7-2	Inbred line	Henan, China	BAAFS	wild type	(Huangzao4 × Wei95) × S901jing
	P178	Inbred line	Beijing, China	BAAFS	wild type	Selected from 78599
	Ye478	Inbred line	Henan, China	BAAFS	wild type	U8112 × Shen5003
	Zheng58	Inbred line	Henan, China	BAAFS	wild type	Selected from Ye478
	Dan340	Inbred line	Liaoning, China	BAAFS	wild type	Baigulû9 × Pod corn
	Qi319	Inbred line	Shandong, China	BAAFS	wild type	Selected from 78599
	C92	Inbred line	Beijing, China	BAAFS	wild type	Chang7-2×Jing24×Lx9801
	Jing724	Inbred line	Beijing, China	BAAFS	wild type	Selected from X1132
	D9H	Inbred line	Beijing, China	BAAFS	wild type	Selected from C8605-2
	B73	Inbred line	America	BAAFS	wild type	BSSS
Zea mays ssp. Mexicana	P1566683	Landrace	America	Zheng et al., 2013	Not analyzed	unkown
	P1566685	Landrace	America	Zheng et al., 2013	Not analyzed	unkown
	P1566691	Landrace	America	Zheng et al., 2013	Not analyzed	unkown
Zea mays ssp. Parviglumis	P1331783	Landrace	America	Zheng et al., 2013	Not analyzed	unkown
	P1331786	Landrace	America	Zheng et al., 2013	Not analyzed	unkown
	M106	Landrace	America	Zheng et al., 2013	Not analyzed	unkown
	P1384061	Landrace	America	Zheng et al., 2013	Not analyzed	unkown
	P1331785	Landrace	America	Zheng et al., 2013	Not analyzed	unkown

Note: BAAFS: Beijing Academy of Agriculture and Forestry Sciences; Other indicates that waxy maize had other mutation in waxy gene, which was different from *wx-hAT*, *wx-D7*, *wx-D10* and *wx-124*;

Supplementary Table S3 Amylopectin content in *wx-hAT* mutant maize

**Accession**	***wx* genotype**	**Starch content (g/100g)**	**Amylopectin content (g/100g)**	**Amylopectin content（%）**
SKN5	*wx-hAT*	65.4	62.7	95.9
SKN6	*wx-hAT*	67.2	63.5	94.5
JN1	*wx-hAT*	66.5	64.5	97.0
HN2	*wx-hAT*	75.3	72.8	96.7
YN1M	*wx-hAT*	61.4	58.2	94.8
JN2	*wx-hAT*	65.5	63.6	97.1
16-585	*wx-hAT*	63.8	61.9	97.0
80482	*wx-hAT*	63.4	61.7	97.3
J6	*wx-D7*	60.4	57.9	95.9
JBN2	*wx-D7*	69.0	67.0	97.1
JKN1	*wx-D7*	68.3	65.8	96.3
JKNS5	*wx-D7*	71.7	68.8	96.0
Zheng58	Wild type			77.27
C92	Wild type			78.88

Supplementary Table S4 Granule bound starch synthase (GBSS) activity in waxy maize and flint maize

**Accession**	***wx* genotype**	**GBSS activity (nmol/min/g)**
YN1M	*wx-hAT*	23.9
80482	*wx-hAT*	28.1
HN2	*wx-hAT*	27.3
SKN6	*wx-hAT*	24.7
SKN5	*wx-hAT*	19.9
JN1	*wx-hAT*	24.5
BN16	*wx-D7*	10.8
HN13	*wx-D7*	12.2
ZHN2	*wx-D7*	9.8
Huaxiangnuo	*wx-D7*	11.2
DN1	*wx-D7*	12.4
Sida30	*wx-D7*	11.9
Zheng58	Wild type	66.7
Chang7-2	Wild type	70.5
D9H	Wild type	73.1
MC01	Wild type	79.3
Jing724	Wild type	76.6
C92	Wild type	71.3

Full-length gels

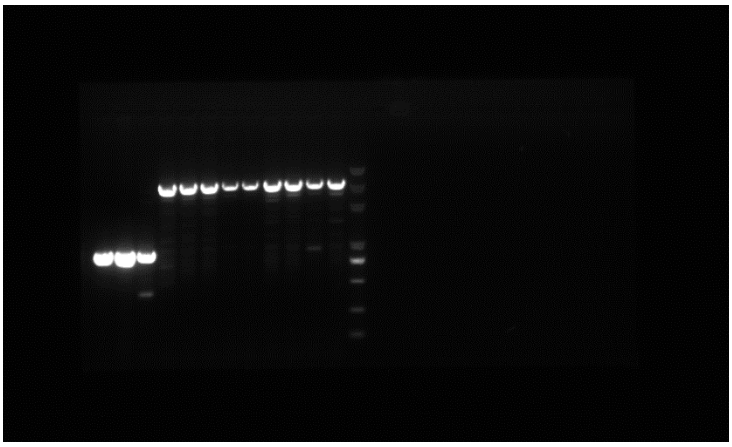

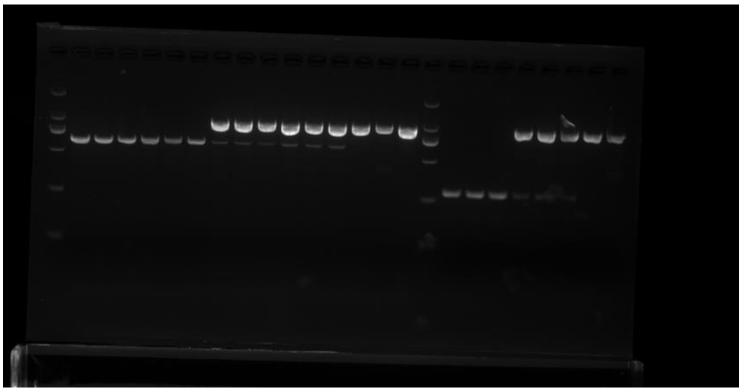

Supplement: Supplementary file 1 — Supplementary Information 1. [file 41598_2020_72764_MOESM1_ESM.pdf]
